# Characterization of microRNA and Metabolite Profiles of Seminal Extracellular Vesicles in Boars

**DOI:** 10.3390/ani15111631

**Published:** 2025-06-01

**Authors:** Jianfeng Ma, Shuang Liang, Siyu Chen, Yuqian Shi, Yu Zou, Lei Chen, Lili Niu, Ye Zhao, Yan Wang, Linyuan Shen, Li Zhu, Mailin Gan

**Affiliations:** 1 State Key Laboratory of Swine and Poultry Breeding Industry, College of Animal Science and Technology, Sichuan Agricultural University, Chengdu 611130, China; 2 Key Laboratory of Livestock and Poultry Multi-Omics, Ministry of Agriculture and Rural Affairs, College of Animal Science and Technology, Sichuan Agricultural University, Chengdu 611130, China; 3 Farm Animal Genetic Resources Exploration and Innovation Key Laboratory of Sichuan Province, Sichuan Agricultural University, Chengdu 611130, China

**Keywords:** boar, semen, extracellular vesicle, miRNA, metabolome

## Abstract

Extracellular vesicles (EVs) contain bioactive substances and mediate a multitude of physiological functions. EVs can be found in most body fluids and are particularly abundant in semen. EVs have the potential to become a biomarker for the quality of boar semen. In this study, EVs were isolated from the semen of relatively young (10 months of age, Y-EVs) and old (30 months of age, O-EVs) duroc boars using ultracentrifugation. The isolated EVs were characterized using a transmission electron microscope, nanoparticle tracking analysis, and Western blotting. MicroRNA (miRNA) profiles and metabolomes were analyzed using high-throughput sequencing and liquid chromatography–mass spectrometry, respectively. The median particle sizes of Y-EVs and O-EVs were 151.3 nm and 162.1 nm, respectively. miR-148a-3p, miR-10b, miR-21-5p, miR-10a-5p, let-7a, etc., were identified as highly enriched miRNAs in seminal EVs of boars. Comparative analysis revealed 41 differentially expressed miRNAs and 132 differential metabolites between Y-EVs and O-EVs. Notably, 18 miRNAs were upregulated in O-EVs, such as miR-339-5p, miR-125a, miR-423-3p, and miR-29c, which were mainly enriched in endocytosis, focal adhesion, and adherens junction. KEGG pathway analysis further indicated that differential metabolites were enriched in glycerophospholipid metabolism. These results provide an insight into the functional roles of seminal EVs.

## Simple Summary

Extracellular vesicles (EVs) contain bioactive substances and mediate a multitude of physiological functions. EVs are widely distributed in body fluids and are particularly abundant in semen. In this study, we successfully isolated EVs from boar semen samples representing two distinct age groups. Through integrated multi-omics analysis, we characterized microRNA and metabolite compositions within these seminal EVs. These results may provide an insight into understanding of the functions of EVs in semen.

## 1. Introduction

Extracellular vesicles (EVs) are membranous vesicles released by most cells into the extracellular space. Based on their biogenesis, EVs are classified into exosomes, microvesicles, and apoptotic vesicles [1]. The size profiles of EV groups overlap significantly, and current isolation techniques cannot effectively separate EVs derived from different biogenesis pathways. Consequently, it is recommended to use the general term “EVs” to describe these heterogeneous vesicles [1]. EVs have been detected in numerous physiological fluids, including saliva [2], breast milk [3], urine [4], and semen [5,6]. Notably, cryo-electron microscopy combined with tomography has revealed multiple EV subtypes in semen [7]. Seminal plasma is known to comprise secretions from the testis, epididymis, and accessory sex glands [8], indicating that semen EVs represent heterogeneous vesicles originating from multiple cells types.

Seminal EVs contain nucleic acids, proteins, and lipids derived from their parental cells. These EVs reflect the physiological status of the male reproductive system and are promising non-invasive biomarkers for predicting semen quality [9]. Research has identified six differentially expressed microRNAs (miRNAs) in seminal EVs from low-vitality and normal-vitality semen [10], demonstrating significant potential as biomarkers for boar semen vitality. Furthermore, Sun et al. characterized small RNA expression patterns in seminal EVs from boar semen containing spermatozoa with cytoplasmic droplets [5], revealing 16 significantly differentially expressed miRNAs potentially associated with this sperm abnormality [5]. In addition to being potential biomarkers, seminal EVs regulate multiple sperm functions. It has been reported that additional EVs added to semen can attach to the sperm head membrane, which could improve sperm membrane integrity [11]. Seminal EVs also improve sperm motility [12], fertilizing capacity [13], and acrosome reaction regulation [14]. Previous studies have shown that semen quality is closely correlated with genetic and environmental factors, and animal age [15]. Increasing evidence suggests that advanced paternal age negatively impacts semen quality, assisted reproductive outcomes, and offspring health [16]. Older males exhibit declining testosterone levels, leading to a reduced total sperm output and increased abnormal sperm rates [17]. However, studies investigating age-related changes in boar seminal EVs remain limited.

Therefore, this study aimed to isolate, characterize, and identify EVs from the semen of young and old boars. Using RNA sequencing and metabolomics, we characterized the miRNA and metabolite profiles of boar seminal EVs, providing new insights into the physiological role of seminal EVs.

## 2. Materials and Methods

### 2.1. Semen Collection and Extracellular Vesicle Isolation

The records of boar semen testing data from a pig farm (Sichuan Province, China) were collected for analysis. All boars were housed in a temperature- and humidity-controlled environment. Semen was collected via a gloved-hand method, with approximately 100 mL obtained from relatively young (10-month-old, *n* = 3) duroc boars and 200 mL from relatively old (30-month-old, *n* = 3) duroc boars. Seminal plasma was obtained by subjecting the semen samples after liquefaction to centrifugation at 1000× *g* for 15 min and then at 3000× *g* for 30 min at 4 °C to remove sperm and cell debris. The supernatant was passed through a 0.22 µm filter for purification; 50 mL of clarified seminal plasma per boar was processed for EV isolation via differential ultracentrifugation. The filtered supernatant was centrifuged at 100,000× *g* for 60 min at 4 °C. The resulting pellet was resuspended in PBS and centrifuged again at 100,000× *g* for 60 min at 4 °C to obtain the EV pellet. The EVs were resuspended in 100 μL cold PBS buffer and stored at −80 °C.

### 2.2. Transmission Electron Microscopys

For transmission electron microscopy (TEM) analysis, EVs were fixed with 2.5% glutaraldehyde for 2 h at 4 °C. A volume of 10 μL of an EV sample was applied to a copper mesh and allowed to stand for 5 min. Excess liquid was absorbed using filter paper. After being blotted and air-dried, the samples were stained with 10 μL 2% uranyl acetate for 1 min. EVs were visualized using an HT7700 transmission electron microscope (Hitachi, Tokyo, Japan).

### 2.3. Nanoparticle Tracking Analysis

The size of EVs was measured by nanoparticle tracking analysis (NTA) using a ZetaView Particle Metrix (PMX-120, Meerbusch, Germany). The instrument was calibrated with 100 nm polystyrene beads. The EV samples were diluted in PBS before NTA. Each sample within every experimental group was subjected to a single NTA measurement.

### 2.4. Western Blotting

An isolated EV sample was lysed with ice-cold RIPA lysis buffer and diluted in SDS-PAGE protein loading buffer. A total of 10 µg of proteins was separated by 10% SDS polyacrylamide gel electrophoresis. Then, the protein was transferred onto polyvinylidene fluoride membranes. A seminal plasma sample was used in negative control samples. The membranes were blocked with experimental quick sealing fluid (AP36L108, Life-iLab, Shanghai, China) for 30 min. Then, the membranes were incubated with anti-TSG101 primary antibody (1:1000 dilution, #381538, ZEN-Bioscience, Chengdu, China), anti-HSP70 primary antibody (1:1000 dilution, #R24633, ZEN-Bioscience, Chengdu, China), and anti-Calnexin primary antibody (1:1000 dilution, #340144, ZEN-Bioscience, Chengdu, China) overnight. HRP–conjugated Goat Anti-Rabbit IgG H&L (1:5000 dilution, WLA023, Wanleibio, Shenyang, China) secondary antibody was incubated for 2 h at room temperature. TBST was used for diluting antibodies and washing. Finally, the blots were visualized with ECL Detection Reagent (36208ES60, Yeasen, Shanghai, China).

### 2.5. Total RNA Extraction, Library Preparation, and Sequencing

Total RNA was extracted using RNAfast200 (Fastagen, Shanghai, China) according to the manufacturer’s instructions. Small RNA sequencing libraries were prepared using TruSeq Small RNA Sample Prep Kits (Illumina, San Diego, CA, USA) using 150 ng of total RNA for input. Libraries were sequenced on an Illumina HiSeq 2500 (Illumina) platform. RNA extraction and sequencing services were provided by the Duolaimi Biotechnology Company (Wuhan, China). The raw sequence data are publicly accessible at https://bigd.big.ac.cn/gsa/browse/CRA018167 (accessed on 25 May 2025).

### 2.6. miRNA Expression Analysis, Target Gene Prediction, and Enrichment Analysis

miRNA data analysis was performed according to the previous workflow [18]. Differential analysis of miRNA expression was performed using DESeq2. Differentially expressed miRNAs were identified using *p* < 0.05 (FDR correction *p*-value) and |log 2 (Fold Change)| > 1. The prediction of miRNA target genes was carried out by OmicStudio tool at https://www.omicstudio.cn/tool (accessing on 9 January 2025) based on TargetScan (5.0) and Miranda (3.3a). GO and KEGG pathway enrichment analysis of target genes was performed using DAVID software (v6.8, https://david.ncifcrf.gov/).

### 2.7. Metabolome Analysis of Seminal EVs

First, the EV samples were used for metabolite extraction. A 500 µL solution (methanol/water = 4:1, *V*/*V*) containing an internal standard was added to 100 µg of EV samples and vortexed for 3 min. The sample was centrifuged at 12,000 rpm for 10 min at 4 °C. A total of 450 μL of the supernatant was transferred and concentrated. A 100 μL solution (methanol/water = 7:3, *V*/*V*) was used to reconstitute the sample and was then centrifuged at 12,000 rpm for 3 min at 4 °C. Supernatants were transferred for LC-MS analysis. All samples were for two LC-MS methods. An aliquot was analyzed on a T3 column (Waters ACQUITY Premier HSS T3, 1.8 µm, 2.1 mm × 100 mm, Milford, MA, USA) with a gradient elution of 0.1% formic acid in water (solvent A) and in acetonitrile (solvent B): 5–20% in 2 min, to 60% in 3 min, to 99% in 1 min, held for 1.5 min, then returned to 5% B in 0.1 min, and held for 2.4 min. Conditions: column temp 40 °C, flow rate 0.4 mL/min, and injection volume 4 μL. Data acquisition was operated using the information-dependent acquisition (IDA) mode using Analyst TF 1.7.1 Software (Sciex, Concord, ON, Canada). The source parameters were set as follows: ion source gas 1 (GAS1), 50 psi; ion source gas 2 (GAS2), 50 psi; curtain gas (CUR), 25 psi; temperature (TEM), 550 °C; declustering potential (DP), 60 V, or −60 V in positive or negative modes, respectively; and ion spray voltage floating (ISVF), 5000 V or −4000 V in positive or negative modes, respectively. The TOF MS scan parameters were set as follows: mass range, 50–1000 Da; accumulation time, 200 ms; and dynamic background subtract, on. The product ion scan parameters were set as follows: mass range, 25–1000 Da; accumulation time, 40 ms; collision energy, 30 or −30 V in positive or negative modes, respectively; collision energy spread, 15; resolution, UNIT; charge state, one to one; intensity, 100 cps; exclude isotopes within 4 Da; mass tolerance, 50 ppm; and maximum number of candidate ions to monitor per cycle, 18.

Raw LC-MS data were converted to mzXML format using ProteoWizard v3.0, followed by peak extraction, alignment, and retention time correction with the XCMS program v1.40.0. Metabolic identification information was obtained by searching the laboratory’s self-built database, integrated public database, AI database, and metDNA. Normalized signal intensities of metabolites were unit variance scaling. OPLS-DA was performed to identify the differential metabolites between Y-EVs and O-EVs, and variable importance in the projection (VIP) was calculated using the OPLS-DA model. In addition, all metabolites were assessed using a Student’s *t*-test. Differential metabolites were determined by VIP > 1, *p* < 0.05, and Fold Change ≥ 1.2. Annotated metabolites were then mapped to the KEGG pathway database. Significantly enriched pathways were identified with a hypergeometric test’s *p*-value for a given list of metabolites.

### 2.8. Statistical Analysis

Statistical analysis of the data was performed using Microsoft Excel software (Microsoft Office Excel 2016) and GraphPad Prism (version 9.5). Pearson’s correlation analysis and data visualization were also performed in GraphPad Prism. A Student’s *t*-test was used to identify differential metabolites between groups. Differences were considered significant at *p* ≤ 0.05.

## 3. Results

### 3.1. Isolation and Identification of Seminal Extracellular Vesicles in Boars

**Figure 1:**
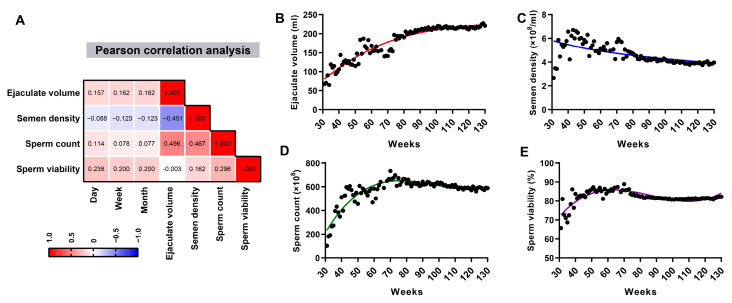
The semen parameters of boars of different ages. (**A**) Pearson correlation between the age and parameters of boar sperm. Statistical analysis of ejaculate volume (**B**), semen density (**C**), sperm count (**D**), and sperm viability (**E**) in different age stages.

Pearson’s correlation analysis was used to investigate the correlation between age and semen parameters of boars. Notably, age was negatively correlated with semen density (r = −0.123) (Figure 1A). Furthermore, statistical results categorized by different weekly age groups indicate that ejaculate volume showed an increasing trend and semen density a decreasing trend with advancing age (Figure 1B,C). The sperm count and viability of boars displayed an initial increase followed by a decrease (Figure 1D,E). Therefore, semen was collected from relatively young (10 months of age) and old (30 months of age) boars for subsequent analysis.

**Figure 2:**
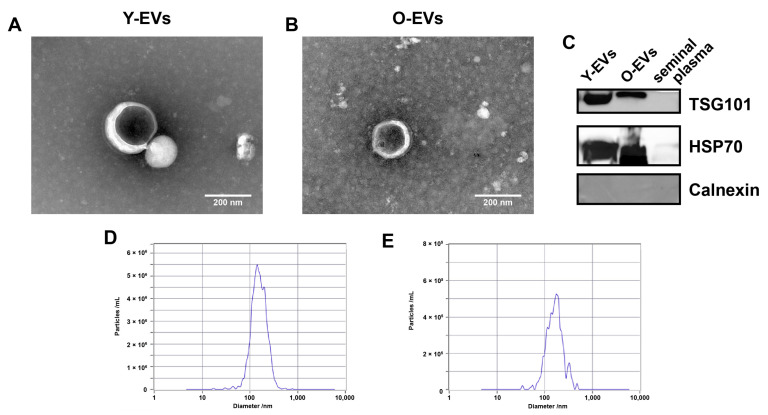
Characterization of seminal extracellular vesicles (EVs). Transmission electron microscopy of seminal extracellular vesicles of young (**A**) and old (**B**) boars. (**C**) Identification of EV proteins markers by Western blot. (**D**,**E**) Vesicle size distribution assessment by nanoparticle tracking analysis.

Extracellular vesicles were isolated from the semen of young (Y-EVs) and old (O-EVs) boars through ultra-high-speed centrifugation. Transmission electron microscopy results showed that the separated Y-EVs and O-EVs had a cup-shaped morphology, characteristic of extracellular vesicles (Figure 2A,B). Western blot analysis indicated that the universal EV markers TSG101 and HSP70 were present in the separated EVs (Figure 2C). NAT analysis results revealed that the median particle size of the separated Y-EVs and O-EVs were 151.3 nm and 162.1 nm, respectively (Figure 2D,E).

### 3.2. Characteristics of miRNA Expression Profiling in Seminal Extracellular Vesicles of Boars

**Figure 3:**
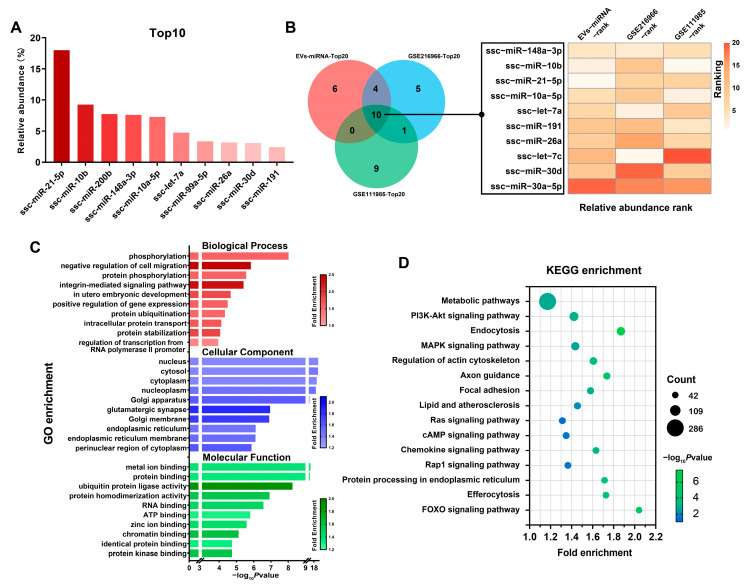
High-abundance miRNA analysis in seminal extracellular vesicles (EVs) of boars. (**A**) Top 10 highly expressed miRNAs in seminal EVs of young and old boars (n = 3 per group). (**B**) Identification of high-expression miRNAs in seminal EVs of boars. (**C**) GO term enrichment analysis of high-expression miRNA target genes. (**D**) KEGG pathway enrichment analysis of high-expression miRNA target genes.

miRNA expression was examined in both groups’ EVs by high-throughput miRNA sequencing. We first investigated the high abundance of miRNAs in EVs from boar semen. High-abundance miRNAs may be candidate markers for EVs in boar semen. Highly expressed miRNAs in EVs of boar semen include miR-21-5p, miR-10b, miR-200b, miR-148a-3p, miR-10a-5p, let-7a, etc. (Figure 3A). We further obtained two other datasets of boar seminal EV miRNAs from the GEO database (GSE216966 and GSE111985). A comprehensive analysis of these data indicates that 10 miRNAs are highly expressed in both these datasets and our results (Figure 3B). Functional enrichment analyses showed phosphorylation, negative regulation of cell migration, integrin-mediated signaling pathway, and in utero embryonic development were the top five enrichments. Meanwhile, within the molecular function category, metal ion binding, protein binding, ubiquitin protein ligase activity, protein homodimerization activity, and RNA binding were the most significantly identified enrichment terms (Figure 3C). KEGG pathway enrichment results showed that the high-abundance miRNAs were enriched in metabolic pathway, PI3K-AKT signaling pathway, endocytosis, etc. (Figure 3D).

### 3.3. Identification of Differentially Expressed miRNAs in Extracellular Vesicles

**Figure 4:**
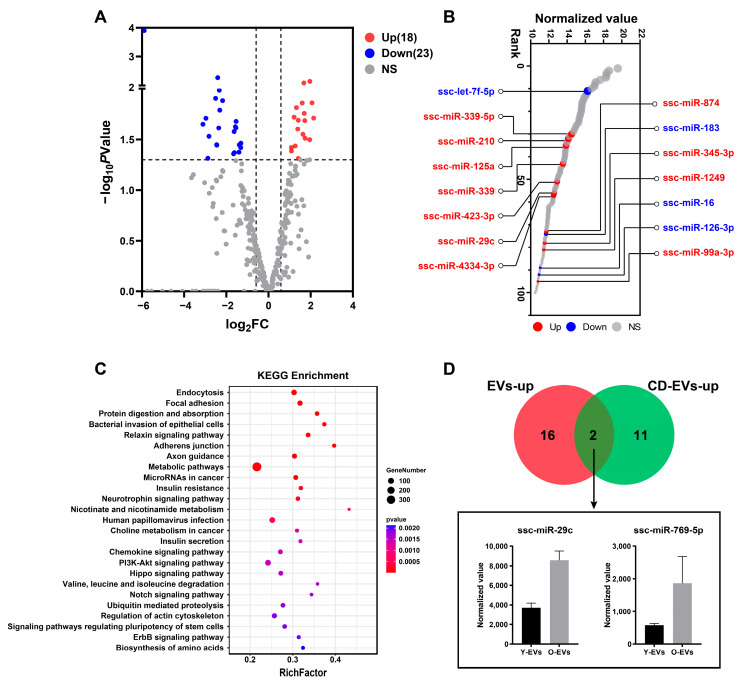
Comparative analysis of differentially expressed seminal EV miRNAs between young and old boars. (**A**) Volcano plot of differentially expressed miRNAs. (**B**) Differentially expressed miRNAs among the top 100 highly expressed miRNAs. (**C**) KEGG pathway enrichment analysis of differentially expressed miRNA target genes. (**D**) ssc-miR-29c and ssc-miR-769-5p were significantly increased in old boar EVs.

A total of 327 miRNAs were detected in the two EV sample groups. Differentially expressed miRNAs were recognized with a Fold Change ≥ 2 and a *p* value ≤ 0.05. A total of 41 miRNAs were differentially expressed between the Y-EV and O-EV groups (Table S1). Of these, 18 were upregulated and 23 were downregulated in the O-EV group (Figure 4A). Further analysis revealed that most of the upregulated miRNAs (11 of 18) were the top 100 most abundant miRNAs (Figure 4B). KEGG enrichment analysis of these upregulated miRNA target genes showed that the related genes were more enriched in endocytosis, focal adhesion, protein digestion and absorption, relaxin signaling pathway, adherens junction, etc. (Figure 4C). Previous studies reported miRNA expression characteristics of boar seminal EVs with cytoplasmic droplets (CD-EVs) [5]. The Venn diagram showed that ssc-miR-29c and ssc-miR-769-5p were significantly upregulated in our results and CD-EVs (Figure 4D). This suggested that ssc-miR-29c and ssc-miR-769-5p may play an important role in boar seminal EVs.

### 3.4. Analysis of Metabolic Features in Extracellular Vesicles of Boar Semen

**Figure 5:**
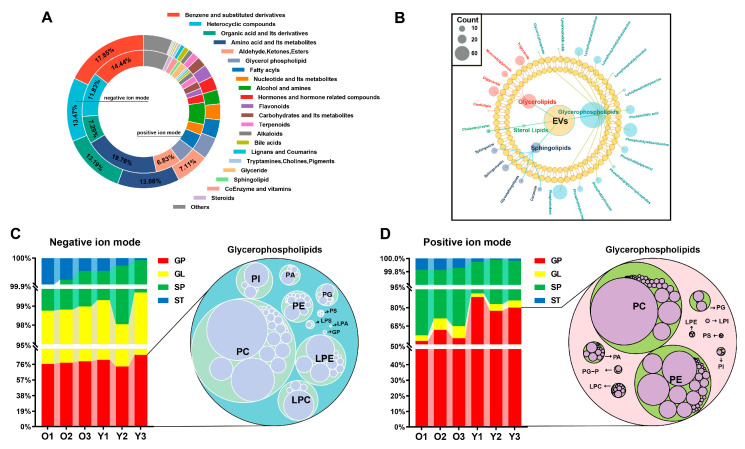
Metabolite profiling of extracellular vesicles (EVs) in boar semen. (**A**) Relative abundance of each metabolite category. (**B**) Classes and counts of lipids identified in EVs of boar semen. Relative abundance of lipid metabolites in negative (**C**) and positive (**D**) ion mode. GP, glycerophospholipids. GL, glycerolipids. SP, sphingolipids. ST, sterol lipids. The bubble plot represents the various subclasses and abundances of glycerophospholipids (*n* = 3 per group).

We next performed a metabolome analysis of EVs to investigate the characteristics of metabolites in boar seminal EVs. A total of 1759 metabolites were identified in negative ion mode and 2798 metabolites were identified in positive ion mode (Tables S2 and S3). All metabolites were detected and divided into 21 chemical classes. In negative ion mode, the highest number of detected metabolites belonged to the benzene and substituted derivatives class, followed by heterocyclic compounds. In positive ion mode, the highest number of detected metabolites belonged to the amino acid and its metabolites class (Figure 5A). Further analysis of the lipid composition of boar seminal EVs revealed that the detected lipid metabolites were divided into four major classes: glycerophospholipids, glycerolipids, sphingolipids, and sterol lipids, comprising a total of 22 subclasses (Figure 5B). Glycerophospholipids were the most abundant lipids in boar seminal EVs, with phosphatidylcholines being the most prevalent subclass. (Figure 5C,D).

### 3.5. Identification of Differential Metabolites in Seminal EVs

**Figure 6:**
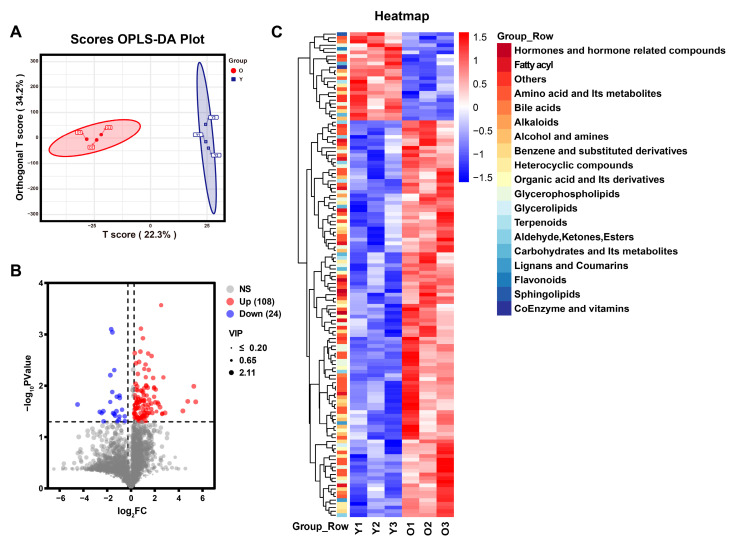
Comparative analysis of differential metabolites of seminal extracellular vesicles between young and old boars. (**A**) OPLS-DA score plot analysis between young and old groups. (**B**) Volcano plot of differential metabolites. (**C**) Cluster heat map of differential metabolites (*n* = 3 per group).

The OPLS-DA model was used to further analyze the differential metabolites between Y-EVs and O-EVs. The OPLS-DA score plot indicated that the Y-EV and O-EV groups were distinctly separated (Figure 6A). A total of 132 differential metabolites (*p* value ≤ 0.05 and VIP ≥ 1) were identified, with 108 upregulated and 24 downregulated metabolites (Figure 6B). Among these, amino acid and its metabolites constituted the largest group (36), followed by glycerophospholipids (16) (Figure 6C). The heat map revealed the relative abundance change of differential metabolites between Y-EV and O-EV groups (Figure 6C).

### 3.6. Functional Enrichment Analysis of the Differential Metabolites

**Figure 7:**
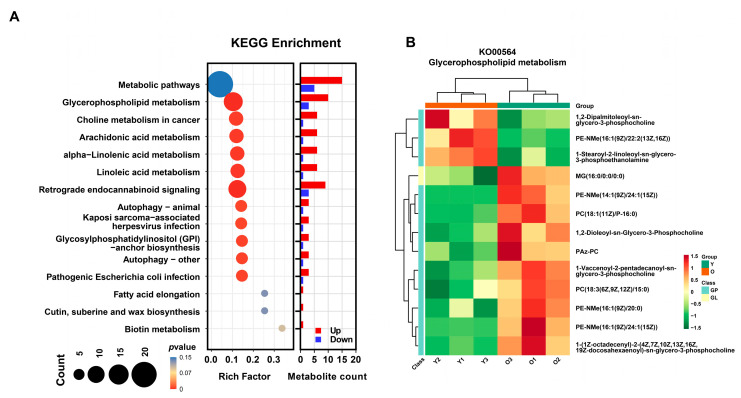
Differential metabolite KEGG enrichments. (**A**) Most significant enriched KEGG pathways. (**B**) Heat map of differential metabolites in glycerophospholipid metabolism pathway.

To further explore the biological functions of differential metabolites, we performed KEGG functional enrichment analysis. Figure 7A demonstrates the significantly enriched KEGG pathways, including metabolic pathway, glycerophospholipid metabolism, arachidonic acid metabolism, retrograde endocannabinoid signaling, etc. Lipids are important components of extracellular vesicles [18]. A total of 13 differential metabolites were significantly enriched in glycerophospholipid metabolism pathways. Figure 7B illustrates the relative levels of these differential metabolites in the Y-EV and O-EV groups.

## 4. Discussion

Extracellular vesicles (EVs) are considered as essential bioactive mediators of cell-to-cell communication, participating in numerous vital biological activities. EVs transfer lipids, nucleic acids, and proteins from donor cells to recipient cells for intercellular communication. Studies have shown that EVs can be found in a variety of body fluids, such as milk [19], urine [20], plasma [21], and semen [5]. EVs are particularly abundant in semen. EVs in semen are heterogeneous vesicles secreted by cells in testes and accessory sex glands [22]. An increasing number of studies have demonstrated that EVs play important roles throughout the male reproductive system. Studies have reported that protein molecules in seminal EVs can promote sperm maturation, such as SLC27A2, EDDM3B, KRT19, and WFDC8 [23]. In addition, seminal EVs contain antioxidant proteins such as ELSPBP1, BLVRA, GPX5, and glutathione transferases that protect sperm from oxidative stress [24]. ATP produced in seminal EVs may modulate mitochondrial metabolism to control sperm motility [12]. Seminal EVs can also reflect the state of male reproductive systems. Studies have reported that miRNAs present in seminal EVs hold promise as non-invasive biomarkers for assessing sperm motility and predicting boar fertility [25]. Age-related variations affect reproductive functions in boars, resulting in marked changes in the biochemical composition of semen [26]. Therefore, in this study, we isolated EVs from the semen of young and older boars to explore age-related changes in seminal EV characteristics.

There are various methods for the isolation of EVs, including ultracentrifugation, density gradient centrifugation, and size exclusion chromatography, with no current gold standard [27]. We used ultracentrifugation, the most widely used method, to isolate EVs from boar semen. Previous studies have isolated extracellular vesicles from the semen of multiple species (humans, pigs, cattle, and rabbit) via ultracentrifugation [6,28,29,30]. Transmission electron microscopy revealed that EVs isolated from boar semen exhibit classic cup-shaped morphology, consistent with ultracentrifugation-derived EVs reported elsewhere [31]. Western blotting analysis confirmed the expression of EV marker proteins TSG101 and HSP70 in the isolated vesicles. In addition, NAT analysis demonstrated EV diameters ranging from 30 to 500 nm, with median particle sizes of 151.3 nm (Y-EVs) and 162.1 nm (O-EVs).

Seminal EVs are known to contain distinctive and specific profiles of highly stable miRNAs. Studies have shown that miRNA expression patterns among seminal EVs, seminal plasma, and spermatozoa are similar [32], indicating that miRNAs within EVs can reflect semen status and possess regulatory functions. Here, we used high-throughput sequencing to analyze miRNA profiles in boar seminal EVs. We identified several highly enriched miRNAs, including miR-21-5p, miR-10b, miR-200b, miR-148a-3p, and miR-10a-5p in boar seminal EVs. Two previous studies reported miRNA profiles of extracellular vesicles in the seminal plasma of Yorkshire boars [33,34]. We integrated two publicly available NCBI datasets (GSE216966 and GES111985) to further analyze the highly enriched miRNAs in boar seminal EVs. Among the top 20 expressed miRNAs in EVs, 10 were shared across all three studies, including miR-148a-3p, miR0-10b, miR-21-5p, etc. These highly enriched miRNAs are potential markers for boar seminal EVs and play indispensable roles in spermatogenesis and homeostasis.

We further identified differentially expressed miRNAs in extracellular vesicles isolated from the semen of young (Y-EVs) and old (O-EVs) boars. In total, 41 differentially expressed miRNAs were identified between Y-EVs and O-EVs. Compared with Y-EVs, O-EVs showed 18 significantly upregulated and 23 downregulated miRNAs. We investigated whether these differentially expressed miRNAs were included in the top 100 expressed miRNAs of boar seminal EVs. Interestingly, most upregulated miRNAs ranked among the top 100 expressed miRNAs, including miR-339-5p, miR-210, miR-125a, miR-29c, miR-423-3p, etc. These miRNA variations may reflect age-dependent changes in seminal EV composition. miR-210-3p in seminal EVs has been reported as a non-invasive biomarker of Sertoli cell damage [35], and Sertoli cells are compromised as aging progresses [36]. Liang et al. reported that miR-125a-5p upregulation in aging males’ sperm correlates with poor sperm DNA integrity, suggesting miR-125a-5p in seminal EVs may serve as an adverse age-related predictor [37]. KEGG pathway enrichment analysis indicated that upregulated miRNA target genes were mainly enriched in endocytosis, focal adhesion, adherens junction, and metabolic pathway, etc. These miRNAs may regulate related germ cell pathways to influence spermatogenesis and maturation. Sun et al. identified 13 upregulated miRNAs (e.g., miR-101, miR-148a-5p, miR-29c, miR-29a-3p, and miR-769-5p) in seminal EVs from semen containing sperm with cytoplasmic droplets [5]. Notably, miR-29c was significantly elevated in O-EVs. Studies suggest miR-29c targets nuclear autoantigenic sperm protein (NASP) [38], which is critical for germ cell development and cell cycle regulation [39].

Extracellular vesicles contain metabolites from donor cells and transfer metabolites to acceptor cells, thereby altering their metabolic status [40]. The metabolic profiles of boar seminal EVs were constructed via untargeted metabolomics. In this study, 2798 and 1759 metabolites were detected in positive and negative ion modes, respectively. EVs were lipid-bound vesicles, making the examination of lipid metabolite composition particularly critical. Lipid metabolites were detected in four categories: glycerophospholipids, glycerolipids, sphingolipids, and sterol lipids, which were further divided into 22 subclasses. Glycerophospholipids were the most abundant lipid metabolites. Notably, their relative levels showed a downward trend in the O-EV group. Studies indicate a positive correlation between EV glycerophospholipid content and their parent cells [40]. Phosphatidylcholines, the predominant glycerophospholipids in EVs [41], were similarly abundant in our findings. OPLS-DA analysis revealed 108 upregulated and 24 downregulated metabolites in O-EVs compared to Y-EVs. KEGG enrichment analysis showed differential metabolites were enriched in metabolic pathways, glycerophospholipid metabolism, arachidonic acid metabolism, etc. A total of 13 differential metabolites were associated with glycerophospholipid metabolism. Glycerophospholipids are key components of sperm membrane [42], regulating osmotic balance [43], sperm motility [44], acrosome reaction, and sperm–oocyte fusion [45]. Seminal EV uptake by sperm modifies membrane lipid composition, enhancing zona pellucida ability [13]. There are limitations to this study that should be acknowledged. The sample size should be increased in future studies to identify more reliable biomarkers of pig semen. Furthermore, functional validation of the identified biomarkers is essential to ensure their reliability.

## 5. Conclusions

In summary, in this study, we characterized the miRNA expression profiles and metabolite profiles of EVs in boar semen samples. This study expanded knowledge of the cargo of boar seminal EVs and contributed to understanding the role of EVs in semen.

## Data Availability

Data are contained within the article, and the research data supporting this study will be shared upon a reasonable request made to the corresponding author.

## Supplementary Materials

The following supporting information can be downloaded at https://www.mdpi.com/article/10.3390/ani15111631/s1, Table S1: List of differentially expressed miRNAs between Y-EV and O-EV groups; Table S2: Metabolites detected in negative ion mode; Table S3: Metabolites detected in positive ion mode.
